# Transitional genomes and nutritional role reversals identified for dual symbionts of adelgids (Aphidoidea: Adelgidae)

**DOI:** 10.1038/s41396-021-01102-w

**Published:** 2021-09-10

**Authors:** Dustin T. Dial, Kathryn M. Weglarz, Akintunde O. Aremu, Nathan P. Havill, Taylor A. Pearson, Gaelen R. Burke, Carol D. von Dohlen

**Affiliations:** 1 Department of Entomology, University of Georgia, Athens, Georgia; 2 Department of Biology, Utah State University, Logan, Utah USA; 3 USDA Forest Service, Northern Research Station, Hamden, Connecticut USA; 4 Present Address: Biology Department, Westfield State University, Westfield, Massachusetts USA

**Keywords:** Microbial ecology, Evolution, Genomics

## Abstract

Many plant-sap-feeding insects have maintained a single, obligate, nutritional symbiont over the long history of their lineage. This senior symbiont may be joined by one or more junior symbionts that compensate for gaps in function incurred through genome-degradative forces. Adelgids are sap-sucking insects that feed solely on conifer trees and follow complex life cycles in which the diet fluctuates in nutrient levels. Adelgids are unusual in that both senior and junior symbionts appear to have been replaced repeatedly over their evolutionary history. Genomes can provide clues to understanding symbiont replacements, but only the dual symbionts of hemlock adelgids have been examined thus far. Here, we sequence and compare genomes of four additional dual-symbiont pairs in adelgids. We show that these symbionts are nutritional partners originating from diverse bacterial lineages and exhibiting wide variation in general genome characteristics. Although dual symbionts cooperate to produce nutrients, the balance of contributions varies widely across pairs, and total genome contents reflect a range of ages and degrees of degradation. Most symbionts appear to be in transitional states of genome reduction. Our findings support a hypothesis of periodic symbiont turnover driven by fluctuating selection for nutritional provisioning related to gains and losses of complex life cycles in their hosts.

## Introduction

Symbiotic associations have repeatedly spurred the diversification of eukaryotic lineages by endowing hosts with novel adaptive traits, unlocking previously unexploited ecological niches [1, 2]. Iconic examples include the intracellular bacterial symbionts of insects that provide essential nutrients to their hosts, notably, in hemipteran insects that feed on a nutritionally unbalanced diet of plant sap. Bacterial symbionts likely facilitated the exploitation of this niche by provisioning essential amino acids (EAAs) and vitamins lacking in plant sap [3].

In many insect groups, strict vertical transmission of symbionts between generations has promoted tight co-evolutionary relationships, but in the process has increased risk of an evolutionary “dead-end”. While symbiosis is adaptive to the consortium, long-term sequestration is costly to symbiont genomes [4]. As a result of greatly reduced effective population sizes, severe population bottlenecks in each generation, relaxed selective constraints, and deletional mutational biases, bacterial symbiont genomes are reduced to the core housekeeping genes and genes essential to the hosts’ survival [5]. Furthermore, strong genetic drift may override purifying selection to spur further losses of genes presumed important to the partnership [6]. When a host’s diet is enriched, relaxed selection on nutritional contributions may facilitate further symbiont gene deletions, locking the insect into its current niche and pushing the consortium further down the evolutionary “rabbit hole” of heritable symbiosis [4]. Escape from this “rabbit hole” is possible only when the host genome can compensate, or when a new symbiont joins the holobiont and supplements or replaces functions of the original one [7, 8].

In support of the symbiotic “rabbit hole”, ancient symbionts of several sap-feeding lineages, such as scales and mealybugs, psyllids, and auchenorrhynchans (cicadas, various hoppers), are mainly found in pairs or even multitudes (e.g., [9–14]). In such dual symbioses, typically a senior symbiont (defined as the older of two obligate nutritional cosymbionts [15]) is accompanied by one (or more) sequentially acquired junior symbiont(s) that completes degraded nutritional pathways. In most dual endosymbioses studied thus far, a senior symbiont provides the majority of genes in EAA metabolic pathways and the junior symbiont(s) supplies the remainder [10, 16–19]. When symbionts occur in multiples, the array of degradative evolutionary forces act on all symbionts, resulting in potential symbiont replacement as genome degradation progresses. Most transitions represent a change in junior symbionts; replacement of senior symbionts is relatively rare, suggesting that time since acquisition is associated with stability [20–25]. This process may be the result of established developmental dependencies and a long history of host adaptations to support eroding symbiont functions [4, 26, 27].

It is not completely understood why most host lineages acquire co-symbionts, while some do not, or why senior symbionts appear to reach a point of stability—and why that stability might break down in some lineages. Studying gains and losses of obligate symbionts in sap-feeding insects is challenging due to the infrequent occurrence of these events and confounding factors such as the diverse feeding habits of the insect hosts. The recently characterized symbionts of Adelgidae (Hemiptera: Aphidoidea) [15, 28–33] offer an opportunity to examine patterns and potential processes of symbiont replacements in a well-defined lineage with similar biology. Comprising approximately 70 described species in five major lineages [34], adelgids feed solely on conifer trees (Pinaceae) and have complex life cycles that include yearly alternation between a primary and a secondary (or alternate) host [35, 36]. Half of their life cycle is spent on spruce (*Picea* spp.) as their primary host plant, where a gall is formed and nutrient-rich parenchyma cells are tapped; the other half is spent on an alternate conifer in one of five other genera, where most species tap relatively nutrient-poor phloem sap. Unlike most other sap-feeding insect lineages of similar or older ages, Adelgidae lack a universal senior symbiont: rather, each adelgid lineage hosts a unique pair of obligate symbionts (as determined from 16 S rRNA gene sequencing and microscopy) [15, 30–32]. This diversity implies that both junior and senior symbionts have been recurrently replaced across the family [15, 30, 31, 33]. These dynamic changes in symbiont composition also align with historical acquisitions of alternate host-plant genera [15].

To date, the only complete, published genomes of adelgid symbionts are those of the hemlock woolly adelgid, *Adelges tsugae*, sampled from the invasive eastern North American population [28]. *A. tsugae* hosts two obligate symbionts: a senior symbiont, “*Candidatus* *Annandia adelgestsuga*”, and a junior symbiont, “*Ca*. Pseudomonas adelgestsugas”. Genomes for both of these organisms bear characteristics of long-term, obligate nutritional symbionts, such as highly reduced, AT-rich genomes missing many core housekeeping genes but retaining complementary genes in nutrient-synthesis pathways. However, in contrast to nutritional-symbiont partners of other sap feeders, symbionts of *A. tsugae* exhibit more balanced contributions to EAA pathways [28]. We previously proposed a hypothesis to explain the frequent replacements of symbionts in Adelgidae, which accounts for the unusually decreased contributions of the senior symbiont in *A. tsugae* [15]. This hypothesis proposes that historical fluctuations in dietary composition during the evolution of host-alternating life cycles may have subjected obligate symbionts to fluctuating selection, thus accelerating gene inactivation in nutrient pathways [28]. If fluctuating selection has been a major driver of symbiont turnover in adelgids, we would expect to find a similar pattern of accelerated degradation in nutrient provisioning pathways of senior symbionts in the other adelgid lineages.

Here, we characterize the genomes of dual symbionts from species of Adelgidae representing the pine, fir, Douglas fir, and larch lineages, and perform comparative analyses including the previously characterized *A. tsugae* from the hemlock lineage. We sought to determine whether these dual symbionts are also nutritional partners, as we have presumed, and whether provisioning profiles are consistent across the family. Thus, we investigated whether genomes of symbionts indicate cooperation in nutrient metabolic pathways, and whether the relatively balanced contributions observed between symbionts of *A. tsugae* are found in other lineages. We further tested whether genome characteristics support the previous hypothesis of symbiont ages and relationships, and whether the dynamics of inferred symbiont gains and losses in this lineage are reflected in their genome-degradation characteristics.

## Methods

### Material acquisition and genome sequencing

Samples selected for sequencing were species from four of the five major conifer-host-associated lineages of Adelgidae (Supplementary Table 1). Species were selected based on the availability of specimens. Samples consisted of pooled individuals from single galls, or from several insects from a single population collected from bark (see Supplementary Table 1). Data from *A. tsugae* [28] were incorporated to represent the fifth lineage from hemlock. The *A. lariciatus* and *P. similis* samples were extracted using the High Pure PCR template kit (Roche Diagnostics, Indianapolis), and treated with DNase-free RNAse (Roche Diagnostics, Indianapolis IN, USA). The *A. piceae, A. kitamiensis*, and *A. cooleyi* samples were extracted using a DNeasy Blood & Tissue Kit (Qiagen Inc., Germantown MD, USA). DNA concentration was quantified with a Qubit fluorometer (Thermo Fisher Scientific, Germantown MA, USA). All samples underwent library construction, and all except the *A. cooleyi* sample underwent Illumina (Illumina Corp., San Diego CA, USA) 150 × 150 paired-end sequencings on either a NextSeq or HiSeq 2500 machine. The *A. cooleyi* DNA was sequenced using Pacific Biosciences (PacBio) (Menlo Park CA, USA) sequencing, after size selection with the BluePippin system (Sage Science, Beverly, MA, USA). Data from *A. kitamiensis* were used only for estimation of genome-wide rates of synonymous (*dS*) substitutions in the symbionts.

### Genome assembly

Raw Illumina reads were quality-trimmed and filtered with Trimmomatic Version 0.36 [37] and the FASTX-toolkit (http://hannonlab.cshl.edu/fastx_toolkit/). Total Illumina reads from *P. similis*, *A. piceae*, and *A. kitamiensis* were quality- and adapter-trimmed using the following parameters with Trimmomatic [37]: ILLUMINACLIP: TruSeq3-PE.fa:2:30:10 LEADING:3 TRAILING:3 SLIDINGWINDOW:4:15 MINLEN:36. *A. lariciatus* reads were filtered with the FASTX-toolkit such that a read was discarded if less than 90% of its bases had Phred quality scores of 30 or more. For all Illumina datasets but *P. similis*, overlapping reads were merged with the paired-end read mergeR (PEAR) [38]. Quality merged and unmerged reads were assembled de novo with SPAdes Version 3.12.0 [39]. The final assemblies for *A. piceae*, *A. kitamiensis*, and *A. lariciatus* symbionts were assembled with the *-merged* flag, while *P. similis* reads were left unmerged and were assembled with the *-meta* flag. All short-read assemblies were performed with k-mers 21, 33, 55, 77, 99, and 127. Assemblies were polished with Illumina reads with Pilon version 1.22 [40]. *A. cooleyi* PacBio reads were assembled and polished with Flye version 2.6 [41]. Symbiont reads were then extracted and isolated from the raw dataset by BLASTn searching against symbiont scaffolds. The resulting reads were reassembled and polished with Flye version 2.6.

Symbiont scaffolds were initially identified on the basis of relative coverage, GC content, and paired-end read mapping. Blobtools v1.01 [42] was used to bin symbiont scaffolds from metagenomic scaffolds into their respective families and to verify that all symbiont sequences were collected. Symbiont scaffolds from SPAdes were fed into SSPACE [43] to create sets of “super scaffolds.” For those that could not be computationally scaffolded or joined with SSPACE, PCR was performed to determine their order and orientation within the genome. Due to the presence of three identical rRNA operons, the “*Ca. Vallotia lariciata*” genome possessed a collapsed repeat. The orientation of “*Ca*. *V. lariciata*” scaffolds were determined by a combination of computational scaffolding, PCR, and shared synteny with “*Ca*. *V. cooleyia*” surrounding breaks. GapPadder [44] was used to extend the lengths of the contigs within the scaffolds. Finally, Pilon was used for misassembly detection and further gap closing.

### Annotation

GC-skew was calculated with GenSkew v.1.0 (http://genskew.csb.univie.ac.at) and used to determine the origin of replication; the origin was designated at the region with the strongest signal where genomes had weak overall GC skew. Initial annotations were performed with the Prokka v1.14 [45] pipeline. Pseudogenes were approximated with Pseudofinder (https://github.com/filip-husnik/pseudofinder/) with default settings and the NCBI nr database (https://blast.ncbi.nlm.nih.gov/). Known bifunctional proteins with at least one intact functional domain were not flagged as pseudogenes. Genes annotated as hypothetical proteins by Prokka were searched against the NCBI RefSeq nonredundant protein (NR) database with BLASTp, and if a function could be assigned, the annotation was adjusted manually. All genomes were checked for insertion-sequence elements with the ISSaga2 web-based interface [46] and verified with BLASTp searches. Amino acid and vitamin-biosynthesis pathways were reconstructed using the BioCyc, EcoCyc, and KAAS databases [47–49]. Sequences for host-support genes were collected from the AphidCyc database and searched against an *A. tsugae* transcriptome (GenBank accession PRJNA242203) and our metagenomic scaffolds to verify the presence. Clusters of orthologous groups (COGs) were determined using the online eggNOG-mapper tool (DIAMOND mapping mode and default choices for other settings) [50, 51]. Statistical tests for comparing means of genes belonging to COG categories for different symbiont types were performed with Analysis of Variance (ANOVA) and Tukey’s Honestly Significant Difference tests. Principal components analysis (PCA) was performed on the proportions of CDS belonging to each of the 26 COG categories relative to the total number of CDS in a given bacterial genome. All statistical analyses were performed with JMP (https://www.jmp.com/en_us/home.html).

### Synteny analysis

Genome-wide synteny was examined between pairs of adelgid symbionts sharing close phylogenetic affiliation. An all-against-all BLAST of amino acid sequences (e-value cutoff = 1e-10) served as input for MCScanX to identify colinear blocks involving more than five genes (parameters: gap_penalty = 5) [52]. Orthovenn2 [53] was used to identify clusters of orthologous genes within symbiont pairs, and output diagrams were modified to reflect the number of genes shared within pairs and those unique to each species. Synteny plots were generated using VGSC 2.0 [54] and modified in Adobe Illustrator (Adobe Corp., San Jose, CA, USA).

### Phylogenetics and molecular evolution

Our initial phylogenetic analysis was performed at the order level (*Enterobacterales*, 158 taxa and 70 orthologs) with genera representing each major proposed family: [55] *Enterobacteriaceae*, *Erwiniaceae*, *Pectobacteriaceae*, *Yersiniaceae*, *Hafniaceae*, *Morganellaceae*, and *Budviciaceae*. The phylogenetic placement of symbionts was inferred with amino acid (AA) sequences using a likelihood-based approach. The phyloSkeleton package [56] was used to collect taxa representing the backbone of the *Enterobacterales* tree. Sequences were downloaded from NCBI and orthologs were determined using a curated list of genomes with HMMER3 [57] (settings: e-value = 0.01, best-match-only) with the Bact109 panortholog gene set included in phyloSkeleton. Species were chosen such that at least one representative from each genus was selected. Additional taxa were included where adelgid symbionts were predicted to cluster based on previous analyses with 16 S rRNA sequences [15, 29, 31]. Three species from the Alphaproteobacteria and Betaproteobacteria were included as outgroups. Orthogroups that did not include target symbionts were excluded and paralogs were manually inspected and removed from the dataset. Orthologous sequences were aligned with Mafft v7.4 [58] (L-INS-I algorithm) and ambiguously aligned regions were trimmed with trimAL v1.4 [59] with the -automated1 option.

Phylogenetic analysis was initially performed with RAxML [60] at the CIPRES Science Gateway (https://www.phylo.org/) with the GTR + G model. However, this method is prone to topological errors due to compositional heterogeneity (e.g., inclusion of AT-rich symbiont genomes) and rapid rates of symbiont evolution. To mitigate this, we recoded our AA datasets by biochemical properties with the Dayhoff-6 recoding scheme and conducted our analysis with PhyloBayes MPI [61], a method that is known to be robust to long-branch-attraction artifacts [62]. The *Enterobacterales* dataset was run with two chains with the options -cat -gtr -dgam = 4. Convergence was tested with the bpcomp and tracecomp commands. The *Enterobacterales* chains did not converge according to bpcomp output even after 30,000 iterations and with 20% burn-in, with maxdiff = 1. However, parameter convergence was reached according to tracecomp statistics, with the maximum discrepancy < 0.3 and the minimum effective size > 50.

After initial placement of *Enterobacterales* symbionts at the order level, we subsampled the taxa from each clade within which an adelgid symbiont clustered, and conducted separate analyses with sets of orthologs common to all taxa identified by Orthofinder [63]. This subsampling allowed the analyses to reach Markov chain Monte Carlo convergence for each tree with higher support and similar topology. Taxa included in the *Burkholderia* tree were manually collected from NCBI. We also conducted a phylogenomic analysis of the *Pseudomonas* clade to place “*Ca*. *Pseudomonas adelgestsuga*”, using a recent phylogeny of *Pseudomonadales* [64] to select representatives of the major lineages for the alignment of orthologous proteins. Orthologous-protein clustering was performed with Orthofinder, and alignment, trimming, and tree construction were performed as above.

We additionally performed a phylogenetic analysis based on 16 S rRNA gene sequences to resolve relationships between the *Serratia*-like adelgid symbionts, *Ecksteinia* and *Gillettellia*. 16 S rRNA gene sequences from *S. symbiotica* were collected from NCBI using previous studies as references for taxon selection [65, 66]. Sequences were aligned with SSU-ALIGN (Nawrocki, 2009) and subsequently inspected in Geneious Prime (version 2019, Biomatters Ltd., Aukland, New Zealand). Ambiguously aligned and divergent sequences were deleted with GBlocks [67] with settings’-b5 = h’ to retain half of positions containing a gap. The resulting alignment was used as input into PhyloBayes with settings -cat -gtr and ran for approximately 40,000 generations per chain (4 chains) until convergence, as tested by bpcomp and tracecomp. Consensus trees were viewed with FigTree (http://tree.bio.ed.ac.uk/software/figtree).

To assess the extent of deviation of intergenic from genic GC content, we grouped intergenic sequences into bins greater than and less than 300 base pairs to differentiate between “normal” intergenic spacers and the large intergenic spacers contributing to the low coding densities of adelgid symbiont genomes. The GC contents of the binned intergenic sequences and CDS were calculated and plotted in R (R Core Team 2020) (https://www.r-project.org/). To determine whether the differences in the genome characteristics of specific symbionts are explained by differences in mutation rate, we estimated their genome-wide rates of synonymous (*dS*) substitutions using Pseudofinder with default settings. Violin plots were made in R using ggplot2 [68].

## Results

### Adelgidae symbionts exhibit wide variation in basic genome characteristics

**Fig. 1:**
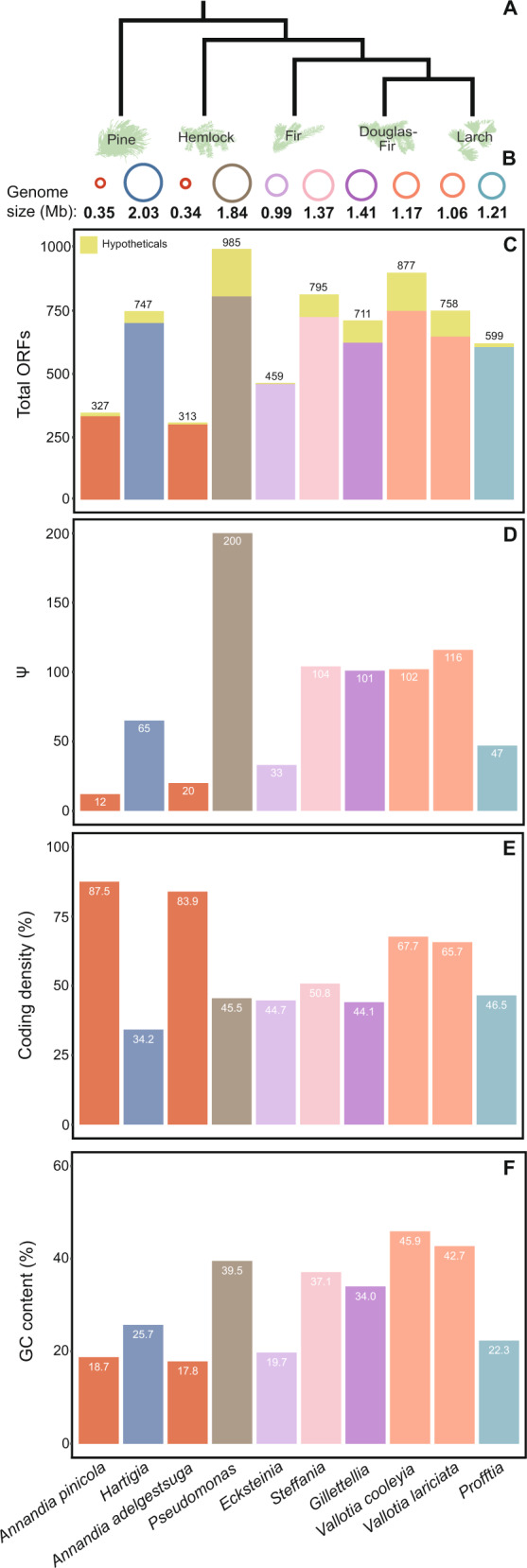
Statistics for adelgid endosymbiont genomes (main chromosomes) sequenced from each major Adelgidae lineage. **A**. Relationships of adelgid species representing the five conifer-host-associated lineages, **B** Chromosome sizes of symbionts (with names indicated at the base of the figure). Circles representing symbiont chromosomes are scaled to indicate size and colored to indicate bacterial lineage (see Fig. 2), **C** Numbers of predicted open-reading frames (ORFs). The proportion of hypothetical proteins composing the proteome is shown in green, **D** Numbers of predicted pseudogenes, **E** The proportion of nucleotides in each symbiont chromosome encoding genes (coding density), **F** The percentage of G or C nucleotides in symbiont chromosomes.

The complete genomes of symbionts from single adelgid species representing four of the five major lineages of adelgids (pine, fir, Douglas fir, and larch) were sequenced and assembled into circular chromosomes to investigate their patterns of genome evolution. We recovered genomes of the two expected symbionts, as characterized previously [29–32], from each adelgid lineage sampled: “*Ca*. *Annandia pinicola*” and “*Ca*. *Hartigia pinicola*” from pine (hereafter, *Annandia pinicola* and *Hartigia*), “*Ca*. *Ecksteinia adelgidicola”* and “*Ca*. Steffania adelgidicola” from true fir (hereafter, *Ecksteinia* and *Steffania*), “*Ca*. Vallotia cooleyia” and “*Ca*. Gillettellia cooleyia” from Douglas fir (hereafter, *Vallotia cooleyia* and *Gillettellia*), and “*Ca*. Vallotia lariciata” and “*Ca*. Profftia lariciata” from larch (hereafter, *Vallotia lariciata* and *Profftia*). To these, we added data from symbionts of *A. tsugae* representing the hemlock lineage, *‘Ca*. Annandia adelgestsuga’ and “*Ca*. Pseudomonas adelgestsugas” (hereafter, *Annandia adelgestsuga* and *Pseudomonas*) [28] to deduce evolutionary patterns and processes across all of Adelgidae. Adelgid symbiont main chromosomes ranged in size from 0.34 Mb to 2.03 Mb, GC contents ranged from 17.8 to 45.9%, numbers of coding sequences (CDS) and pseudogenes varied from 313 to 985 and 12 to 200, respectively, and coding densities varied from 87.5 to 34.2% (Fig. 1), reflecting a dynamic history of genome evolution and symbiont replacement throughout the adelgid family.

We also recovered putative plasmid sequences in each dataset during the assembly and binning process. For datasets from *A. cooleyi* and *A. lariciatus*, plasmids were assigned to the *Vallotia* genomes (pAcVc for *A. cooleyi* and pAlVl for *A. lariciatus*) due to an abundance of genes that shared the highest sequence similarity with relatives of *Vallotia* (see below). The pAcVc and pAlVl plasmids were 61 and 67 kbp in size, possessed 38.2% and 41.8% GC content, and had 28 and 36 CDS (12 and 4 pseudogenes), respectively. Six additional plasmids recovered for the *A. piceae* and *P. similis* datasets (named pAp1 through −3 and pPs1 through −3) could not be assigned to symbionts because their genes shared identity with diverse bacteria (Supplementary Table 2). However, from *A. piceae*, the GC contents of two plasmids (pAp2 and pAp3) were more similar to *Ecksteinia* while the other (pAp1) was more similar to *Steffania*. These plasmids generally encoded genes involved in replication, recombination, metabolism, translation, transport, protein folding, and gene transfer.

### Adelgidae symbionts originate from diverse bacterial lineages

**Fig. 2:**
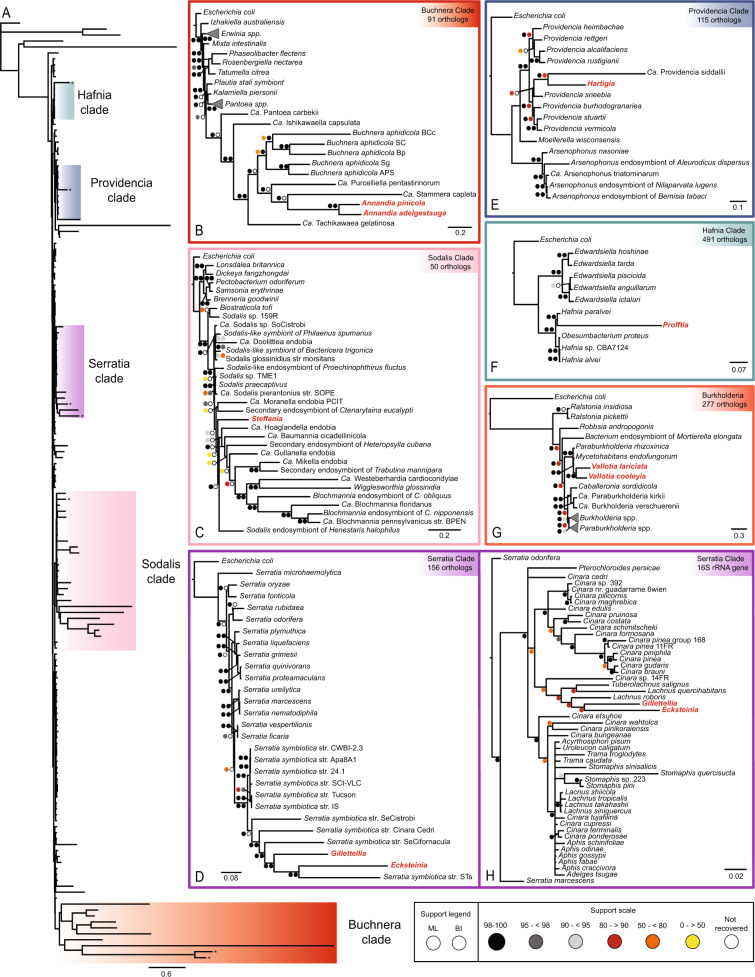
Phylogenetic relationships of adelgid endosymbionts within each major Adelgidae lineage. **A** Multi-gene Bayesian phylogeny of the *Enterobacterales* with 158 taxa inferred from 70 orthologs recoded under the Dayhoff-6 scheme. Colors indicate clades of taxa at the family level to which adelgid symbionts belong, **B**–**F** Maximum likelihood (shown) and Bayesian phylogenies of individual clades in which adelgid symbionts clustered in (**A**) using orthogroups where all taxa were represented, **G** Multigene maximum likelihood (shown) and Bayesian phylogeny of the *Burkholderiales*. H. Bayesian 16 S rRNA gene phylogeny of the *Serratia symbiotica* clade; *Serratia symbiotica* from aphid species are denoted by aphid species names. Adelgid symbiont names are shown in red. Support values are listed as bootstrap replicates supporting nodes for maximum likelihood trees (ML) and percentage posterior probabilities for Bayesian inference phylogenies (BI). Bayesian support values are from analyses conducted on Dayhoff-6-recoded datasets, except for C, where sequences were left uncoded.

Phylogenomic analysis of the *Enterobacterales* placed adelgid symbionts within diverse and well-supported lineages (Fig. 2A). Within *Erwiniaceae*, the two *Annandia* species, along with ‘*Ca*. Purcelliella pentastirinorum’ and “*Ca*. Stammera capleta” formed a clade sister to *Buchnera* spp. (Fig. 2B). *Steffania* clustered within the *Sodalis* clade in *Pectobacteriaceae* (Fig. 2C). *Ecksteinia* and *Gillettellia* grouped within the *Serratia symbiotica* clade, but were not sister taxa as found in a previous 16 S rRNA gene analysis [32]. Instead, they were separated by *Serratia symbiotica* from the giant willow aphid *Tuberolachnus salignus* (STs) (Fig. 2D). Greater taxon sampling of the *S. symbiotica* clade in the 16S rRNA gene phylogeny placed *Ecksteinia* and *Gillettellia* as sister taxa, but with low support (Fig. 2H). *Hartigia* clustered within the *Providencia* clade (Fig. 2E), a genus of free-living bacteria and insect-associated symbionts, as sister to “*Ca*. Providencia siddallii”, a symbiont of glossiphoniid leeches that provisions B vitamins to its host [69]. *Profftia* fell within *Hafniaceae* (Fig. 2F), a group of facultatively aerobic bacteria commonly isolated from the gastrointestinal tract of humans and animals [70].

Phylogenomic analysis of the *Burkholderiales* placed the two betaproteobacterial *Vallotia* species as clustering with two endosymbionts of the fungus *Rhizopus microsporus*, *Mycetohabitans endofungoum* and *Paraburkholderia rhizoxinica* (Fig. 2G). Previous work hypothesized that *Vallotia* was derived from these fungal symbionts, although only one species, *Vallotia tarda* from the larch lineage, was included in the analysis [71]. Our two additional *Vallotia* genomes, representing the larch and Douglas-fir lineages, clustered within the *Mycetohabitans* clade, further strengthening this hypothesis. Phylogenomic analysis of the *Pseudomonas* (*Pseudomonadales*) representatives revealed that the closest relatives to *P. adelgestsugas* were *P. edaphica* and *P. salomonii* within the *P. fluorescens* clade, the largest and most diverse *Pseudomonas* group [64] (Supplementary Fig. 1). Together, these results show that while several symbionts of major adelgid lineages arose through independent acquisitions, others arose through introductions in a common ancestor of multiple host lineages.

### Pairs of symbionts with phylogenetically affiliated progenitors vary in the degree of similarity in genomic content and architecture

**Fig. 3:**
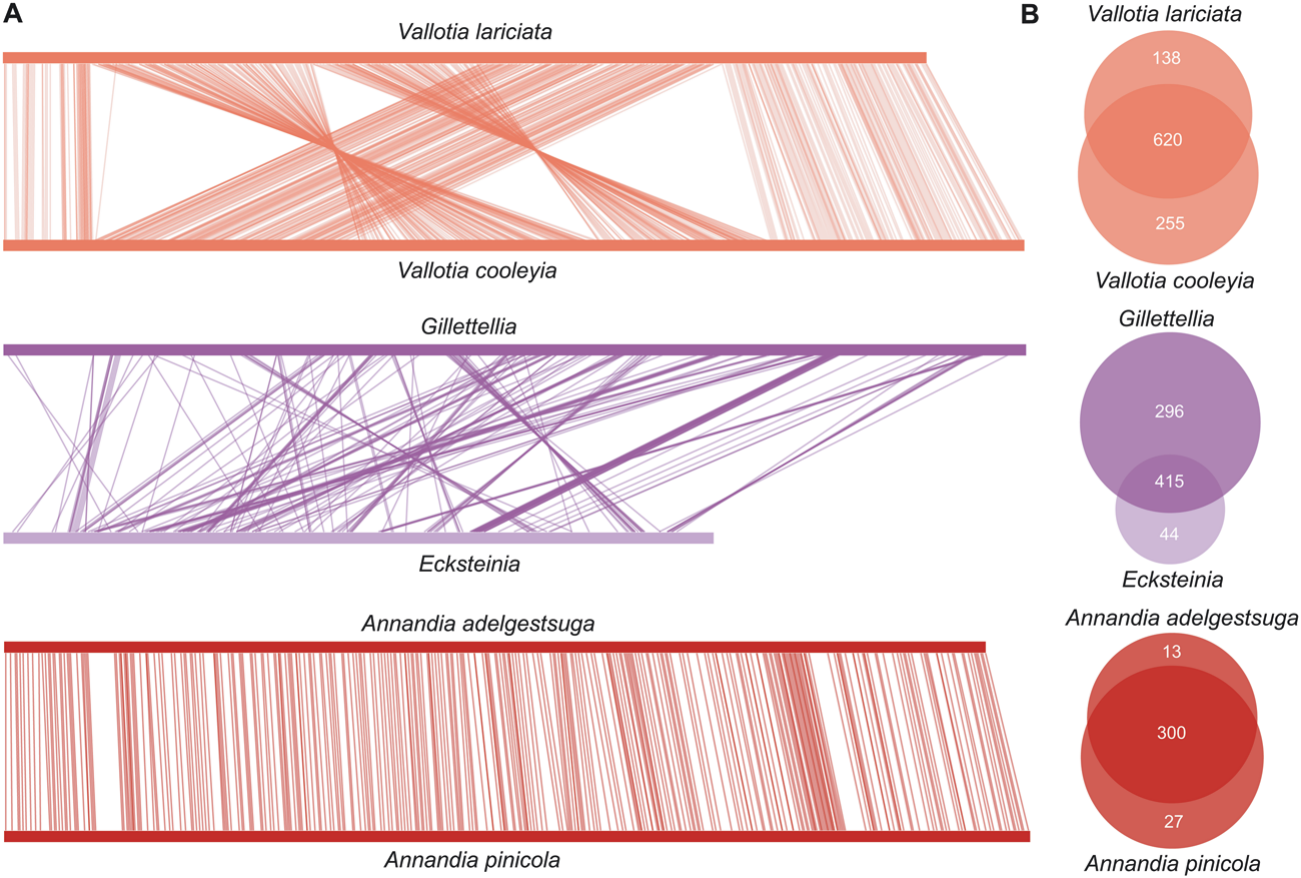
Genome structure and shared genomic content of related adelgid symbionts. **A** Genome synteny comparisons of adelgid symbionts with similar ancestors. Each line between two chromosomes links a pair of colinear genes found in syntenous blocks of a minimum of five genes. Bars representing genomes are scaled within synteny plots of each pairwise comparison. The *Annandia* genomes are perfectly colinear, the *Vallotia* genomes exhibit several rearrangements, and *Ecksteinia* and *Gillettellia* exhibit little synteny. **B** Venn diagrams displaying shared and unshared protein-coding genes between each pair. Circle overlaps represent the numbers of orthologs shared between species, and numbers outside overlaps show unshared genes (paralogs or singletons). Circles are sized to approximate the number of genes in each genome.

Analysis of synteny between related symbiont pairs revealed varying levels of conservation in gene order. Synteny between the two *Annandia* species was highly conserved, with the majority of both genomes retaining the same gene content and order (Fig. 3). Synteny between the two *Vallotia* species was lower; they shared five large conserved blocks but also showed some rearrangements and inversions. Despite both being derived from within a lineage of *Serratia-*like symbionts, *Ecksteinia* and *Gillettellia* genomes differed dramatically in gene order, genome size, and numbers of shared CDS. *Annandia pinicola* and *Annandia adelgestsugas* share nearly their entire genetic repertoire, while the *Vallotia* genomes each possess more unique genes. Many unique genes in the *Vallotia* genomes are hypothetical (Fig. 1C), but the average GC content of these genes is more similar to non-hypothetical CDS than to intergenic spacers, implying that they are experiencing some degree of selective constraint. The similarities between the *Annandia* and *Vallotia* pairs are indicative of single-acquisition events, while the genomic differences between *Ecksteinia* and *Gillettellia* are suggestive, but not conclusive, of independent origins.

### Adelgidae symbionts cooperate to produce all essential amino acids and certain vitamins, but contributions vary across symbiont pairs

**Fig. 4:**
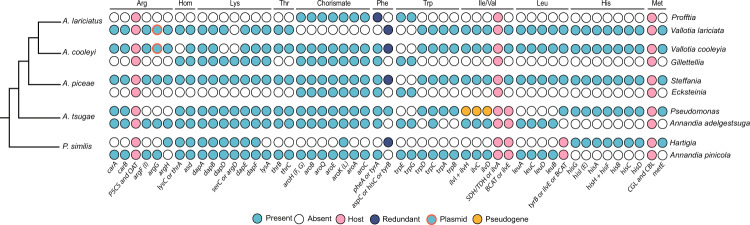
Nutritional complementation in the production of essential amino acids and precursors in cosymbionts from each adelgid lineage. Genes encoding enzymes involved in biosynthetic pathways are shown as column names. Standard abbreviations for genes are used. Gene presence or absence in adelgid symbiont main chromosomes and plasmids is shown, along with contributions from host (adelgid) genes when genes with similar functions are missing from symbiont genomes. Genes are indicated as redundant when there exists more than one gene or gene copy that can complete the pathway.

We found evidence of nutritional complementation in each set of cosymbionts for each adelgid species. With probable contributions from the host [72, 73], each consortium possesses genes to complete pathways for all essential amino acids (EAA). While the lysine pathway of *A. lariciatus* symbionts is missing two genes, identical gene losses have occurred in endosymbionts of related insects [73, 74], suggesting this pathway may be supplemented by unknown host or symbiont enzymes. Altogether, our data suggest a complex history of gene retention and loss in nutritional pathways. Fig. 4 shows the presence and absence of genes for canonical EAA biosynthesis pathways and key precursors (homoserine is required for threonine and methionine and chorismate is required for phenylalanine and tryptophan). In many cases, one of the two symbionts contributes most or all genes for the biosynthesis of a given EAA. For example, arginine, threonine, isoleucine, valine, leucine, and histidine are often produced entirely or mostly by one symbiont. In other cases, the biosynthesis of an EAA is almost always achieved cooperatively by the dual symbionts, for example, with the exception of the symbionts of *P. similis*, the tryptophan pathway has been divided such that one symbiont performs the first two rate-limiting steps with trpEG and the other symbiont performs the following steps with trpDCAB. The complement of genes in other EAA pathways is less consistent. Cosymbionts of *P. similis*, *A. tsugae*, and *A. cooleyi* are fully (or nearly fully) redundant in the lysine pathway, while only *Steffania* and *Vallotia* contribute lysine genes in *A. piceae* and *A. lariciatus*, respectively. Cosymbionts of *A. tsugae*, *A. piceae*, and *A. cooleyi* possess fully redundant chorismate-biosynthesis pathways, while in *P. similis* and *A. lariciatus*, only one of the two symbionts is capable of synthesizing chorismate. The contributions of *A. piceae, A. cooleyi*, *A. lariciatus* and *P. similis* co-symbionts to EAA-biosynthesis are far less evenly distributed than in *A. tsugae*. In all symbiont pairs but those of *A. tsugae*, one symbiont encodes the majority of EAA biosynthesis genes. *Gillettellia* has very few EAA biosynthetic capabilities that are not encoded by its *Vallotia* partner; the only relevant genes it possesses are trpEG, dapD, serC, and lysC/thrA for tryptophan, lysine, and homoserine. *Profftia* cannot produce any EAA on its own; this symbiont encodes two genes for tryptophan biosynthesis (trpEG), is the sole contributor of chorismate, and cooperates with its *Vallotia* partner to make phenylalanine. *Ecksteinia* contributes the least to EAA biosynthesis, encoding only two genes for tryptophan biosynthesis (trpEG) and genes for chorismate and phenylalanine that are redundant with its partner. Beyond amino acid provisioning, all symbionts retain at least some genes to produce vitamins and co-factors, but these pathways are largely incomplete (Supplementary Fig. 2). Intriguingly, pseudogenized thiamine genes in a plasmid recovered from our *A. piceae* dataset share the greatest sequence similarity with thiamine genes of “*Ca*. Hamiltonella defensa” and “*Ca*. Erwinia haradaeae” symbionts of Lachninae aphids (Supplementary Table 2). This may imply a common origin for these genes and that they are horizontally transferred between symbiont lineages relatively often [75]. Moreover, each *Vallotia* plasmid contains *argG* and *tyrB* (encoding components of the arginine and phenylalanine EAA pathways, respectively), which may contribute to the maintenance of this plasmid in the genome. The plasmid of *Vallotia cooleyia* contains functional *thiC* and *thiD* (encoding components of the thiamine pathway), while these genes have been pseudogenized in the plasmid of *Vallotia lariciata*. Overall, while adelgids of the hemlock lineage hosts symbionts that are fairly balanced in their nutritional contributions, adelgids in the fir, Douglas fir, larch, and pine lineages rely on one symbiont far more heavily than the other.

### Coding and noncoding content of Adelgidae symbiont genomes reflects a range of ages and stages of degradation

**Fig. 5:**
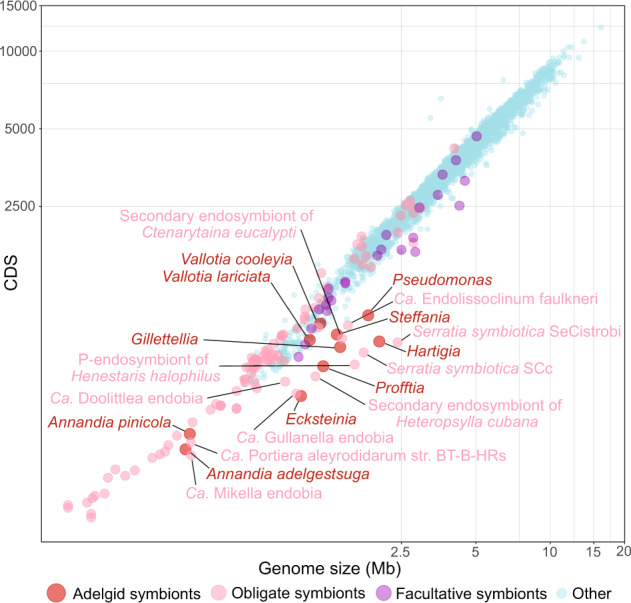
Relationship between genome size and total number of CDS in genomes of adelgid symbionts and other representative bacteria. The names of symbionts with an unusual genome size relative to numbers of CDS (coding density) are indicated.

A notable feature in Adelgidae is the concentration of symbionts with genomes containing large intergenic spacers (IGS). In ancient obligate symbionts and many other bacteria, typically there is a tight correlation between the genome size and number of protein-coding sequences in bacterial species, i.e., genomes are gene-dense with only short spacer regions (Fig. 5). The few genomes with lower-than-typical coding densities correspond to recently derived symbionts from disparate lineages. While both *Annandia* and *Vallotia* symbionts fall within the expected correlation, the other adelgid symbionts have larger genomes than predicted given their numbers of protein-coding genes (Fig. 5). We detected no insertion sequences in these genomes; thus, the large genome sizes relative to the number of coding sequences in adelgid symbionts are not due to the proliferation of repetitive sequences or mobile elements. These low-coding densities, ranging from 34.2 to 50.8% (Supplementary Table 3), are indicative of symbionts in an intermediate stage of genome degradation, where the larger intergenic regions represent sequences that were once intact genes but have not yet been purged from the genome [76]. *Annandia* and *Vallotia* genomes are more typical of the high-coding densities observed in many ancient obligate symbionts [76–78].

**Fig. 6:**
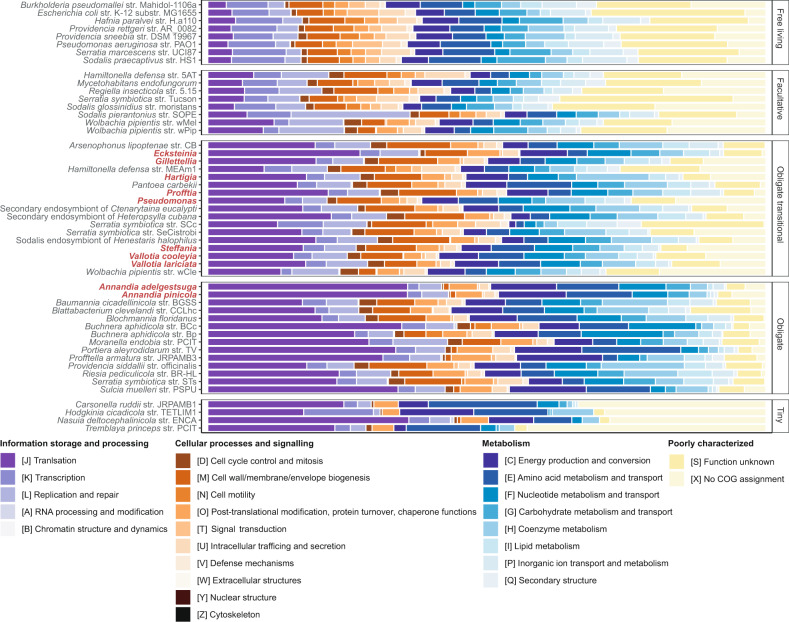
Classification of protein-coding sequences into general COG functional categories. Uncategorized sequences were assigned to a custom category X. Taxa were grouped into free-living, facultative, obligate–transitional (>0.7 Mbp), obligate (<0.7 Mbp), and tiny–obligate (<0.2 Mbp) genome categories. Adelgid symbionts are shown in red text. Free-living and facultative bacteria related to adelgid symbionts were chosen for comparison.

To gain further insight into the relative degree of genome degradation of adelgid symbionts, we determined the proportion of genes belonging to each COG category and made comparisons to other bacteria in various stages of genome degradation (Fig. 6; Supplementary Fig. 3) [76]. Genomes of *Annandia*, the hypothesized ancestral symbiont of Adelgidae, were most like obligate symbionts, which typically retain a large proportion of genes in translation (category J) and lose many poorly characterized genes (categories S and X) compared with free-living bacteria and facultative symbionts. *Ecksteinia* also experienced greater retention in category J relative to all other symbionts, except *Annandia*, with *Profftia* having the (marginally) next highest. The two *Annandia* species, *Ecksteinia*, and *Profftia*, experienced the greatest proportional reductions in poorly characterized genes, with the remaining adelgid symbionts appearing most like facultative or free-living symbionts in this respect. Genes underlying central cellular processes, as compiled in Moran and Bennett (2014) [79] and Bennett et al. (2014) [80], showed varying degrees of retention (Supplementary Fig. 4). Both *Annandia* species and *Ecksteinia* showed the greatest degree of gene loss in DNA-replication initiation, cell division, phospholipid and fatty acid synthesis, and peptidoglycan synthesis. Overall, these comparisons suggest that, relative to other adelgid symbionts, *Annandia*, *Ecksteinia,* and *Profftia* have patterns of gene retention most similar to bacteria in the obligate symbiont category.

**Fig. 7:**
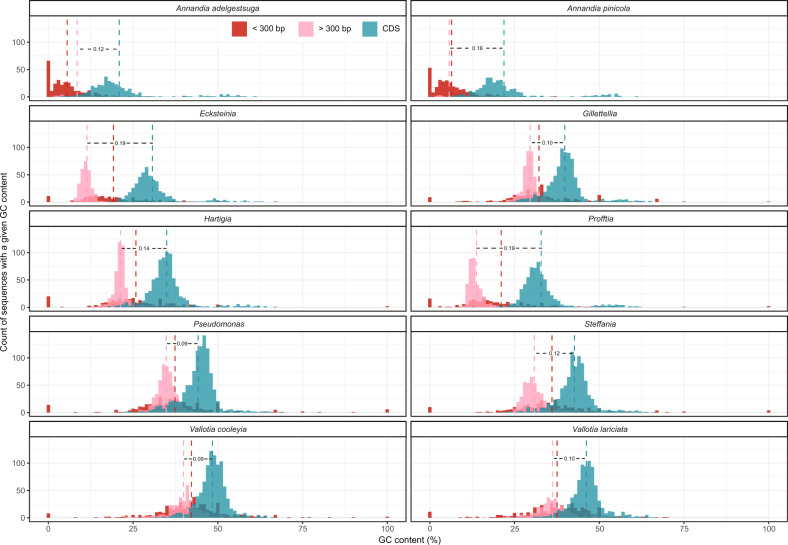
Distributions of GC percentages in short intergenic sequences (<300 bp, red), long intergenic sequences (>300 bp, pink), and CDS (blue). Differences between CDS and long intergenic GC percentages are shown between lines indicating distribution means.

We calculated the GC contents of all the CDS and IGS separately for each symbiont genome (Fig. 7). We divided IGS into those that are small in size (representing IGS typical of bacteria with normal average coding densities) and large in size, which presumably represent “fossils” of genes that have accumulated sequence changes but not enough large deletions to reduce the genome to normal coding density. The *Annandia* genome was typical of many obligate symbionts with very few large IGS. For the remaining symbionts, the GC peaks for large intergenic sequences differed from those of the CDS, with differences most pronounced in *Profftia* and *Ecksteinia* and least pronounced for the two *Vallotia* species. Furthermore, we searched for homology within the IGS to quantify the degree of IGS divergence from ancestral genes. The extent of detectable homology to known genes in the IGSs is reflected in the numbers of pseudogenes flagged as “no predicted ORF” by Pseudofinder due to BLASTx hits in intergenic regions (Supplementary Table 3). The two *Annandia* species, *Ecksteinia*, *Profftia*, and *Hartigia* appear to have the least amount of homology in their IGS relative to other symbionts. We found that *Vallotia* and *Profftia* have similar *dS* values (Supplementary Fig. 5), suggesting that *Profftia’s* more extreme departure of GC content in CDS vs. IGS and lack of intergenic homology is not due to differences in mutation rate. The high relative AT content in IGS and only trace homology to known genes are features expected of symbionts in relatively more advanced stages of genome reduction as a consequence of longer periods of internment [81–83].

## Discussion

Previous phylogenetic studies of rRNA gene sequences have revealed a high degree of diversity and a dynamic evolutionary history of symbiosis within the Adelgidae [15, 29–33]. These studies identified unique pairs of symbionts in each of the five major adelgid lineages, which were consistent within each lineage. These bacteria were inferred to be nutritional partners on the basis that their hosts’ alternating, nutritionally unbalanced diet required supplementation, and that the symbiont genomes of *A. tsugae* from the hemlock lineage confirmed a pattern of nutritional supplementation and complementation [28]. The present study sought to address prevailing questions concerning the evolution of symbiosis within the Adelgidae, including (i) whether dual symbionts in the other adelgid lineages are nutritional and show similar provisioning patterns, (ii) whether the relationships previously proposed are supported by genomic data, and (iii) whether dynamic gains and losses of symbionts are correlated with genome-degradation characteristics. We found that adelgid symbionts are indeed obligate nutritional partners, but their genomes vary greatly in the degrees of degradation and patterns of nutritional cooperation, consistent with a dynamic history of gains and losses. This work raises intriguing questions regarding the underlying drivers of genome degradation and the role that host ecology plays in symbiont gene loss and turnover.

### The Adelgidae possesses an unusually high concentration of symbionts in transitional states of genome degradation

Genomes of adelgid symbionts exhibit a broad spectrum of degradation, varying from tiny and compact, to larger and with low-coding densities typical of genomes in an intermediate stage of reduction. While some possess little-to-moderate AT bias (e.g., *Steffania*, *Vallotia*, *Pseudomonas*, and *Gillettellia*) and share COG distributions similar to other intermediately reduced genomes, other genomes with low-coding densities possess properties more similar to symbionts in an advanced state of genome reduction (e.g., *Profftia* and *Ecksteinia*). Typical of most ancient obligate symbionts, all adelgid symbionts have lost tRNA genes and contain only a single functional rRNA operon, except *Vallotia*, which has three identical rRNA operons. While examples of symbionts with abundant, large intergenic sequences exist (e.g., [84, 85]), we have found an unprecedented concentration of symbionts, from diverse lineages, with these IGS in the adelgids. The transition to endosymbiotic life is thought to be accompanied by wide-ranging gene inactivations caused by relaxed selection on genes redundant with the host and a reduction in effective population size [76]. While previous studies of a handful of transitioning symbionts have documented a delay between gene inactivation and subsequent deletion that is resolved over time, many adelgid symbionts are in a transitional state, despite varying in relative age and ancestry. These features are suggestive of high turnover rates, potential genome redundancy at the onset of symbiosis, and inefficient selection, and may occur convergently due to shared life-cycle characteristics of hosts.

### Support for a hypothesis of symbiont gains, losses, and relationships

Within the framework of symbiont gains and losses proposed by Toenshoff et al. (2014) [31] and expanded upon by von Dohlen et al. (2017) ([15] Fig. 6), *Annandia* was posited as the ancestral symbiont of Adelgidae [28]. *Annandia* was replaced by the common ancestor of *Ecksteinia* and *Gillettellia*, and the latter was joined by the junior symbiont *Vallotia*. *Gillettellia* (senior) and *Vallotia* (junior) diversified with the Douglas-fir lineage. *Vallotia* displaced the *Gillettellia/Ecksteinia ancestor* as the senior symbiont before diversification of the larch lineage. *Hartigia*, *Pseudomonas*, *Steffania*, and *Profftia* were all hypothesized to have entered as junior symbionts before the diversification of each of their respective host lineages (pine, hemlock, true fir, and larch, respectively). A major goal of the present study was to test this scenario using information from symbiont genome data, and from phylogenetic analyses with broader taxonomic representation incorporating new data from GenBank.

As in our original hypothesis, our analyses clearly support the placement of *Annandia* as the ancestral symbiont of adelgids. The genomes of *Annandia* symbionts from pine and hemlock lineages are highly similar, and phylogenetic analyses place them as sister taxa with strong support. This ancestral symbiont was most likely acquired sometime in the late Cretaceous period in the Adelgidae stem lineage [15, 31]. The high level of synteny shared by these two symbionts indicates that they became stable before the diversification of their hosts, as is frequently seen in other ancient, obligate symbiont-host partnerships [21, 22]. *Hartigia* has a large genome with very-low-coding density, similar to *Serratia* junior symbionts in *Cinara* aphids [84], supporting its placement as the junior symbiont in the pine lineage. As the junior companion to *Annandia adelgestsuga*, *Pseudomonas adelgestsugas* is younger than *Annandia*, but most certainly acquired in the stem hemlock lineage [15, 32].

Previously, we hypothesized that *Ecksteinia* and *Gillettellia* shared a single progenitor in the ancestor of the fir, Douglas fir, and larch lineages that split ~65 million years ago [15, 30, 86]. This ancestral symbiont would have codiversified with its hosts in the fir and Douglas-fir lineages and been replaced in the larch lineage. Our current results appear to contradict this interpretation, while suggesting a different evolutionary scenario. Our phylogenomic analyses showed with strong support that, although closely related, *Gillettellia* and *Ecksteinia* were not sister taxa. It is conceivable that this topology was an artifact of signal-confounding long-branch attraction; however, our methods were designed to account for this. The topology also might have resulted from reduced taxon sampling imposed by the limited genomes available for comparison, a possibility supported by the sister relationship of *Ecksteinia* and *Gillettellia* in our species-rich 16 S rRNA gene tree (albeit with low support). Other evidence to support independent acquisitions of these symbionts includes that *Ecksteinia* and *Gillettellia* share little synteny relative to the *Annandia* or *Vallotia* pairs; moreover, they differ greatly in genome size, GC content, and coding capacity. We cannot completely discount that *Ecksteinia* and *Gillettellia* are sister taxa that diverged rapidly before the loss of mobile elements that allowed for lineage-specific rearrangements, as observed in *S. symbiotica* in aphids [87]. However, on the totality of current evidence, we argue that the most likely explanation is that these symbionts arose from independent acquisitions from different *Serratia*-like ancestors. Indeed, *Serratia symbiotica* and *Sodalis*-like bacteria are known to form obligate symbiotic relationships repeatedly in other systems (e.g., [88, 89]).

In contrast to our previously proposed hypothesis, our genomic data suggest that *Profftia*, not *Vallotia*, may be the oldest symbiont of the stem larch + Douglas-fir lineage. *Profftia* possesses more signatures of long-term sequestration than either *Vallotia* or *Gillettellia* with regard to coding capacity, AT bias, the extent of intergenic sequence degradation, and redundancy in nutritional pathway genes with its partner. *Profftia* possesses fewer coding sequences than *Gillettellia* or either *Vallotia* spp., and its average genomic GC content is more comparable to the clearly ancient *Annandia* and *Ecksteinia*. Long-term obligate symbionts have often lost most of their rRNA operons, with many only possessing one or two [90]. *Profftia* retains a single isolated 16 S rRNA gene and adjacent 23 S and 5 S rRNA genes, while *Vallotia lariciata* encodes three identical operons. Genes encoding the tricarboxylic acid cycle (TCA) are often lost in long-established symbionts and are present in facultative and more recently obligate symbionts [79, 91, 92]. *Vallotia lariciata* retains a nearly complete set of TCA cycle genes, while *Profftia* has retained few. Large intergenic regions representing once-intact genes are expected to accumulate neutral mutations at a clock-like rate, resulting in sequences that steadily become more AT rich over time compared with genic sequences that are constrained by purifying selection [81–83]. Among all adelgid symbionts, the degree of departure of intergenic from genic GC is most extreme in *Profftia* and *Ecksteinia*. Moreover, *Profftia* has an average intergenic length similar to *Gillettellia* and approximately twice that of either *Vallotia*, but possesses less detectable homology in its intergenic sequences than *Gillettellia* or *Vallotia* spp. Redundancies in nutritional pathways are thought to be eliminated 30–60 million years after codependency develops [88]. *Profftia* and *Vallotia lariciata* share no EAA-pathway genes, whereas *Vallotia cooleyia* and *Gillettellia* have redundancies in homoserine, lysine, and chorismate, suggesting the former partnership is comparatively older. Our *dS* comparisons of *Profftia* and *Vallotia* suggest that these differences in genome characteristics are not explained by differences in substitution rates caused by differential loss of DNA-repair genes or replication times. Rather, with our existing data, these differences are best explained by the unequal lengths of time these bacteria have been evolving as obligate endosymbionts, with *Profftia* sequestered the longest. We note that factors other than age of association may influence the relative degree of symbiont genome degradation within an adelgid species. For example, differences in the severity of bottlenecks experienced by each symbiont during vertical transmission could change the strength of genetic drift affecting stochastic gene loss. Future work to explore this idea could quantify the titers of dual symbionts provisioned to eggs, as well as potential changes in symbiont numbers throughout the life cycle.

**Fig. 8:**
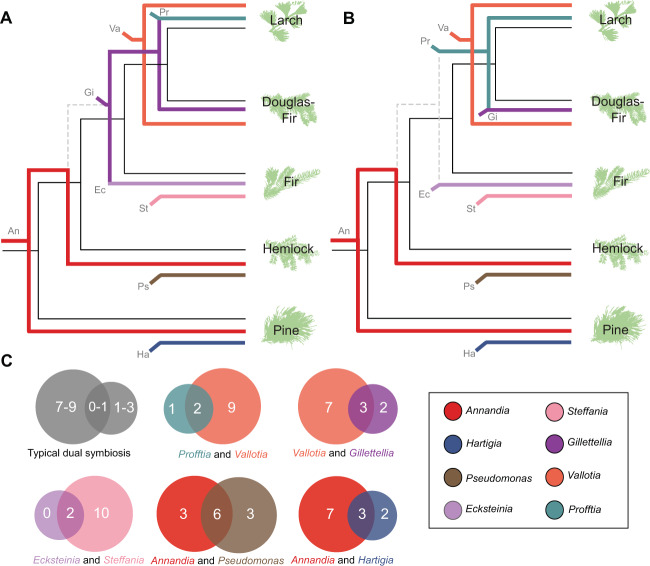
Possible scenarios of symbiont replacements during the evolution of Adelgidae. **A** Hypothesis proposed in von Dohlen et al.([15]). **B** Current hypothesis based on the results of this study. Two-letter codes and colored lines indicate acquisition and retention of specific symbionts. **C** Venn-like diagrams showing the relative contributions of each symbiont to synthesizing EAA and precursors (homoserine and chorismate). In each pair, hypothesized senior symbionts are represented by circles on the left and juniors on the right; age relationships are based on B. Numbers where circles overlap indicate EAA or precursors that symbionts cooperate to produce. In pathways where partial redundancies exist between symbionts, the symbiont contributing genes for the full pathway is counted as the sole contributor.

We propose the following scenario as the most parsimonious, given our genomic data (Fig. 8). *Annandia* was acquired deep within the stem lineage of the Adelgidae, analogous to the history of aphids and their *Buchnera* symbionts [93]. *Hartigia* and *Pseudomonas* were acquired as junior symbionts in the pine and hemlock lineages, respectively. *Annandia* was lost in the stem lineage of the fir, Douglas fir and larch lineages and *Ecksteinia* was acquired either before the divergence of the fir lineage or soon thereafter. We cannot say exactly when *Ecksteinia* was acquired, except that it was almost certainly before *Steffania*. *Profftia* was likely acquired in the stem lineage of the Douglas-fir and larch lineages before *Vallotia*, and was replaced by *Gillettellia* in the Douglas-fir lineage. In each case, we cannot say whether symbionts were ever hosted in triplicate during adelgid evolution.

The history of symbiont replacements in adelgids is unusual among sap-feeding insects in three respects. First, adelgids have replaced both senior and junior symbionts multiple times over their comparatively short (<100 MY) and species-poor history. Other much older and more diverse lineages have generally coevolved with a single senior symbiont, e.g., *Buchnera* in aphids (160 + MY), *Sulcia* in Auchenorrhyncha (~270 MY), *Evansia* in Coleorrhyncha (~250 MY), *Carsonella* in psyllids (~250 MY), and *Portiera* in whiteflies (~100–200 MY) [23, 94–97]. Second, both senior and junior symbionts of adelgids generally derive from unrelated bacterial lineages, whereas in other insects, junior symbionts often derive from the same or related bacterial lineages, e.g., *Serratia* in aphids; *Sodalis*-like symbionts in psyllids and mealybugs [19, 89, 98]. Third, adelgid dual symbionts vary in the proportion of nutrient provisioning undertaken by each partner, while in other insects the senior symbiont typically provides the majority [17–19, 99, 100]. We recognize that while adelgids may stand out as unusual at present, future research may uncover similar replacement patterns in cooperative nutritional symbioses. This is likely the case in Coccoidea; symbionts in this group are diverse [101, 102] but the nutritional status of many is yet to be explored.

### Atypical nutritional provisioning profiles are consistent with the idea that fluctuating selection on nutritional requirements is a driver of symbiont turnover

While partnered symbionts consistently possess complementary genes in nutrient biosynthesis pathways, we find that specific gene breakdown in these pathways does not follow a common pattern across adelgid lineages or between junior versus senior symbionts. Adelgid symbionts are clearly interdependent, requiring their partners (and hosts) to produce the metabolites necessary for survival of the insect–bacterial consortium. That no two adelgid symbiont pairs are identical in the particulars of how and what they provision to their insect host further supports that the process of individual gene loss is at least partially stochastic.

Losses in EAA-provisioning capabilities of symbionts are sometimes linked to trophic shifts of the insect host to more nutritious diets. One example includes the loss of *Buchnera* arginine-biosynthesis genes in aphids that feed inside galls [103]. Several aphid and leafhopper symbionts have lost specific EAA pathways associated with a shift to feeding on phloem sap rich in the same EAA [21, 104, 105]. In more extreme cases, trophic shifts are related to a loss of nutritional symbionts entirely, e.g., in certain leafhoppers feeding on nutrient-rich parenchyma [23]. These examples demonstrate that changes in an insect’s diet can alter selective forces acting to maintain nutrient provisioning by symbionts, or even the symbionts themselves, resulting in degradation of the symbiosis.

The unusually high turnover of obligate symbionts in Adelgidae might be explained by a history of fluctuations in dietary quality related to complex life-cycle evolution and gall formation [15]. Nutrient-partitioning strategies among the five symbiont pairs investigated here suggest that senior symbionts incur accelerated losses of nutritional capabilities as compared with senior symbionts in other sap-feeding insects, leading to frequent acquisitions of junior symbionts and losses of senior symbionts. Most other hemipteran insects studied in depth (reviewed in [79]) lack the complex life cycles of adelgids and thus do not experience regular and repeated bouts of fluctuations in plant-sap quality. They also exhibit stable relationships with senior symbionts that play consistent, majority roles in EAA provisioning. In contrast, in adelgids, the senior symbiont is the primary provider in only two lineages (pine and Douglas-fir), shares nearly equal responsibility in hemlock, and provides very few EAA genes in the fir and larch lineages. During periods of relaxed selection, when adelgids feed on nutrient-rich galls on spruce hosts, accelerated gene inactivations in the nutrient-provisioning pathways should occur indiscriminately between these dual symbionts, allowing either symbiont to reduce its nutrient-provisioning responsibilities or to acquire a partnership-ending mutation. Periods of increased selection, when adelgids feed on nutrient-poor phloem of alternate hosts, can occur yearly for populations that regularly alternate between spruce and other conifers, or can last for up to thousands of years for populations on alternate hosts separated from spruces due to glacial cycles or invasions to new environments [36, 106]. Thus, the varied nutrient provisioning contributions of adelgid symbionts may be a consequence of historical and contemporary fluctuations in host dietary quality that spurs symbiont genome decay and turnover [15, 28].

## Supplementary information

Supplementary Information
