# Supplementary material for: Transitional genomes and nutritional role reversals identified for dual symbionts of adelgids (Aphidoidea: Adelgidae)
Source: ISME J. 2021 Sep 10;16(3):642–54. doi: 10.1038/s41396-021-01102-w (PMC8857208; doi:10.1038/s41396-021-01102-w)
Supplement: Supplementary file 1 — Supplementary Information [file 41396_2021_1102_MOESM1_ESM.pdf]

Supplementary Table 1. Collection and identification information for adelgid samples sequenced in this study.

**Adelgid species**	**Voucher ID**	**Conifer lineage**	**Collection information**
Adelges lariciatus	Ad13-081	Larch (*Larix*)	CANADA: British Columbia: Vernon: Kalamalka Seed Orchard. 21 xi 2013. Ex: *Picea sitchensis*, from gall. J. Corrigan, collector
A. kitamiensis	NH18-608-01	Larch (*Larix*)	JAPAN: Yamanashi: Kiyosato Hokuto-shi; 10 xiii 2018. Ex: *Picea maximowiczii*, from gall. S. Shiyake, collector
A. cooleyi	19-005CV	Douglas-fir (*Pseudotsuga*)	USA: ID: Franklin County, Mile 11.5 on ID-36; 23 vii 2019. Ex: *Picea coloradensis*, from gall. C. von Dohlen, collector
A. piceae	Ad18-003.6	True fir (*Abies*)	USA: UT, Mantua; 12 vi 2018. Ex: *Abies fraser*i, from bark. C. von Dohlen, collector
Pineus similis	Ad13-065	Pine (*Pinus*)	CANADA: British Columbia: Vernon: Kalamalka Seed Orchard. 21 xi 2013. Ex: *Picea sitchensis*, from gall. J. Corrigan, collector

Supplementary Table 2. Genetic characteristics and genic contents of plasmids recovered from *A. piceae* and *P. similis* assemblies.

Host adelgid	Plasmid node	Plasmid name	Size	%GC	Assembled into circular sequence	Gene	Intact/ Pseudogene	Top blast hit	Perc. Ident	evalue	ref
A. piceae	NODE_3533	pAp1	19,437	33.5	Y	thiazole synthase thiG	Pseudogene	*Candidatus* Hamiltonella defensa	72.5	9.67E-28	ref|WP_158368048.1|
						2-iminoacetate synthase thiH	Pseudogene	*Candidatus* Hamiltonella defensa	55.495	7.33E-54	ref|WP_187306980.1|
						ABC transporter permease	Pseudogene	*Serratia rubidaea*	72.619	6.84E-37	ref|WP_126533423.1|
						autotransporter domain-containing protein	Intact	*Serratia symbiotica*	62.331	9.75E-157	gb|NIH12260.1|
						helix-turn-helix domain-containing protein	Intact	*Salmonella enterica*	68.027	2.15E-69	gb|EAQ6300284.1|
						Autotransporter beta-domain superfamily protein	Intact	*Candidatus* Erwinia haradaeae	39.623	1.07E-150	emb|VFP86476.1|
						thiamine phosphate synthase thiE	Pseudogene	*Candidatus* Hamiltonella defensa	53.846	7.20E-06	ref|WP_158380130.1|
						ATP-dependent RecD-like DNA helicase	Pseudogene	*Candidatus* Doolittlea endobia	66.901	7.06E-67	emb|CUX96934.1|
A. piceae	NODE_10959	pAp2	4616	21.5	Y	lipoyl synthase lipA	Intact	*Serratia symbiotica*	69.792	7.38E-153	gb|NIH12524.1|
						ribose-5-phosphate isomerase RpiA	Intact	*Serratia symbiotica*	72.558	7.77E-113	gb|NIG88331.1|
						RepB family plasmid replication initiator protein	Intact	*Providencia rettgeri*	67.117	3.02E-108	ref|WP_196535193.1|
						tRNA-leu	Intact	*Providencia heimbachae*	93.98	2.00E-25	CP028384.1
						Grx4 family monothiol glutaredoxin	Intact	*Candidatus* Arsenophonus triatominarum	69.412	8.78E-40	ref|WP_063656336.1|
A. piceae	NODE_8735	pAp3	6680	21.4	Y	Hsp20 family protein	Intact	*Buttiauxella warmboldiae*	60.897	4.38E-68	ref|WP_124022293.1|
						ABC transporter substrate-binding protein	Intact	*Sodalis*-like symbiont of *Philaenus spumarius*	48.945	3.80E-57	gb|OZI14334.1|
						replication associated protein RepA1	Intact	*Candidatus* Hamiltonella defensa	75.618	5.32E-155	ref|WP_174889681.1|
P. similis	NODE_5623	pPs1	7561	25.9	N	replication initiation protein	Intact	*Arsenophonus nasoniae*	60.064	6.05E-134	ref|WP_135679345.1|
						autotransporter outer membrane beta-barrel domain-containing protein	Intact	*Candidatus* Regiella insecticola	65.909	5.53E-147	ref|WP_006706193.1|
P. similis	NODE_8772	pPs2	3236	28.3	N	Autotransporter beta-domain superfamily protein	Intact	*Candidatus* Erwinia haradaeae	38.786	6.03E-155	emb|VFP88765.1|
P. similis	NODE_11021	pPs3	1988	26.5	N	TraY domain-containing protein	Intact	*Candidatus* Hamiltonella defensa	67.273	1.73E-18	ref|WP_100103797.1|

Supplementary Table 3. Basic genome (main chromosome) characteristics and GenBank accessions of adelgid symbionts. Accessions for *Vallotia* symbionts list the main chromosome plus plasmid.

Bacterium	GenBank Accession	Genome size (bp)	Average intergenic length	Intergenic sequences > 300 bp	Total ORFs (CDS)	Hypothetical CDS	Overall GC	Total Ψ	Ψ (no predicted ORF)*	tRNA	rRNA	Coverage
‘*Ca.* Annandia adelgestsuga’	CP026513	334,746	108.9	16	313	8	17.80%	20	7	29	1,1,1	17
‘*Ca.* Annandia pinicola’	CP045876	348,580	84.1	10	327	11	18.70%	12	4	28	1,1,1	1430
‘*Ca.* Ecksteinia adelgidicola’	CP045239	980,458	1116.7	289	459	4	19.70%	33	10	33	1,1,1	50
‘*Ca.* Profftia lariciata’	CP045925	1,206,213	1046.3	352	599	12	22.30%	47	24	35	1,1,1	29
‘*Ca.* Profftia kitamiensis’	CP080506	1,185,617	1238.6	338	578	6	24.7%	38	20	34	1,1,1	58
‘*Ca.* Gillettellia cooleyia’	CP047043	1,413,195	1147.7	401	711	91	34.00%	101	60	37	1,1,1	115
‘*Ca.* Steffania adelgidicola’	CP045889	1,369,541	867.3	433	795	87	37.10%	104	62	40	2,1,1	130
‘*Ca.* Hartigia pinicola’	CP048208	2,034,350	1882.3	434	747	51	25.70%	65	37	35	1,1,1	118
‘*Ca.* Vallotia lariciata’	CP047398-CP047399	1,064,389	508.5	316	758	87	42.70%	116	70	43	3,3,3	264
‘*Ca.* Vallotia cooleyia’	CP047396-CP047397	1,174,085	466.2	345	877	112	45.90%	102	61	43	3,3,3	1049
‘*Ca.* Vallotia kitamiensis’	CP080504-CP080505	1,105,254	534.7	306	800	66	43.4%	62	36	43	3,3,3	187
‘*Ca.* Pseudomonas adelgestsugas’	CP026512	1,835,598	1063.2	519	985	177	39.50%	200	163	38	1,1,1	35

*This sub-category of pseudogenes contains those that have no predicted ORFs, but nevertheless have homology to protein-coding sequences in the NR database when the region between flanking genes is translated into 6 frames (https://github.com/filip-husnik/pseudofinder/)

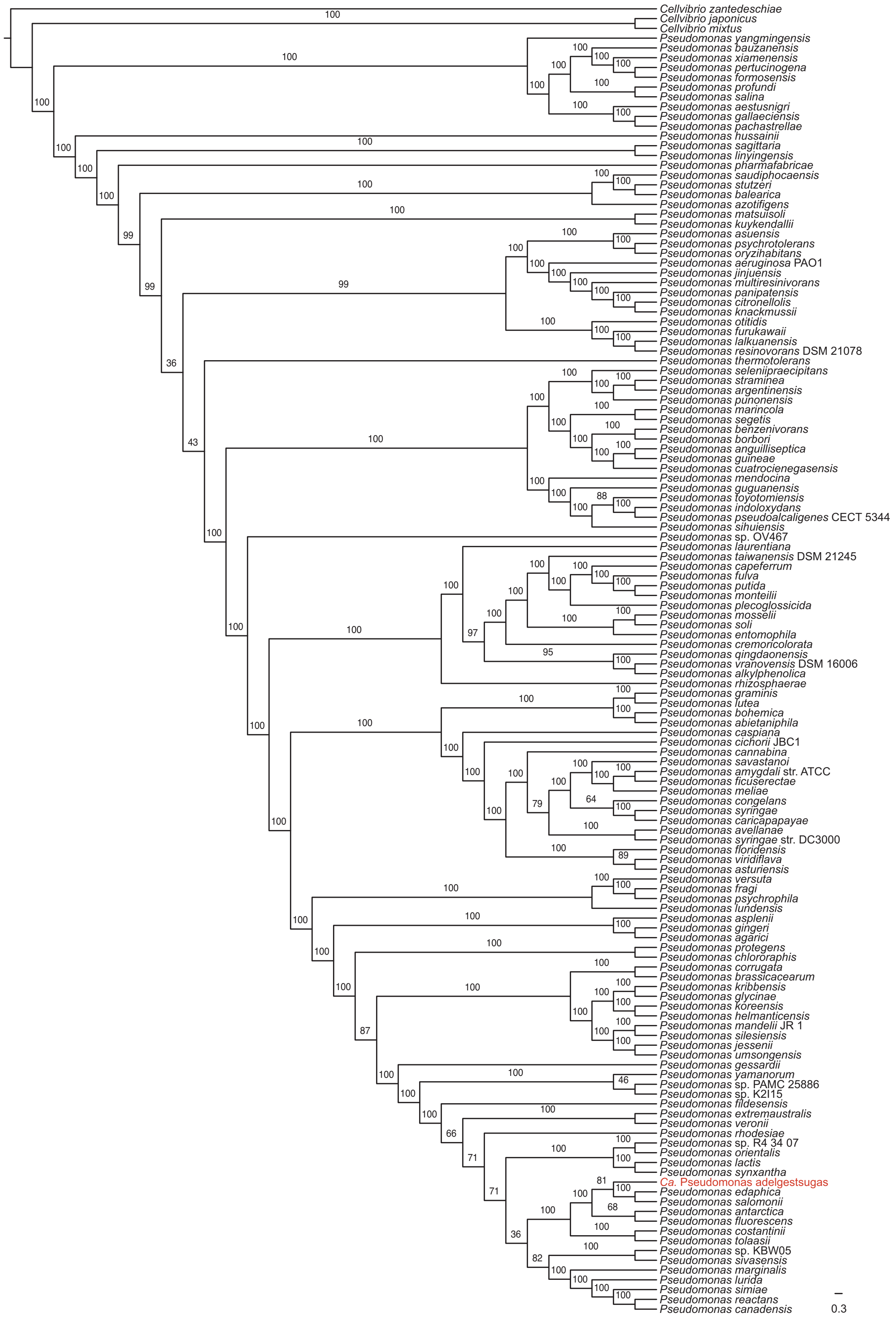

Supplementary Figure 1. Phylogenetic relationship of *Pseudomonas adelgestsugas* from *A. tsugae* within the *Pseudomonadales*. Maximum likelihood phylogeny using 351 single-copy orthologs. *P. adelgestsugas* is shown in red. Support values are based on 1000 bootstrap replicates.

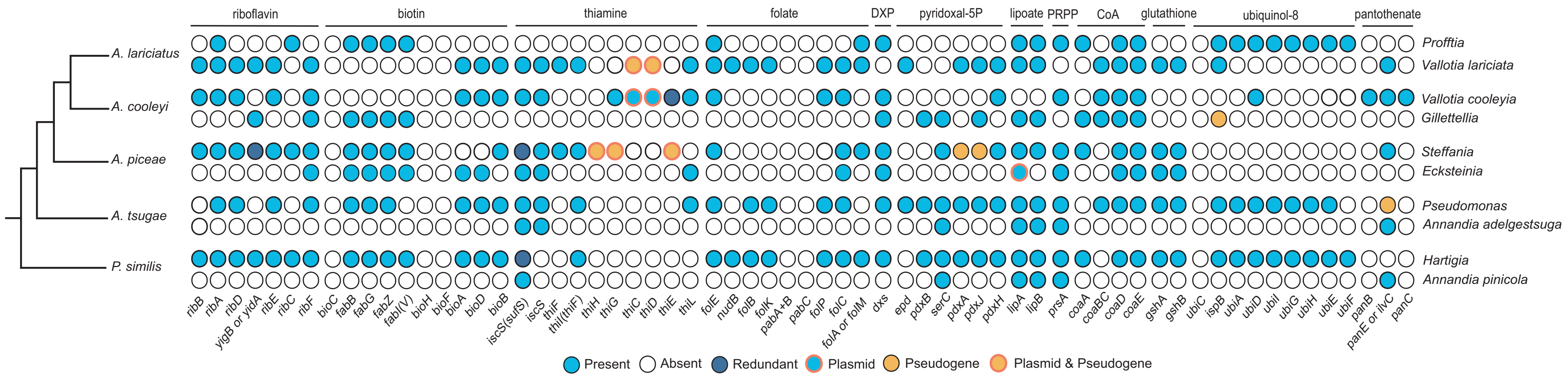

Supplementary Figure 2. Nutritional complementation in production of vitamins for co-symbionts from each adelgid lineage. Genes encoding enzymes involved in biosynthetic pathways are shown as column names. Standard abbreviations for genes are used. Genes are indicated as redundant when there exists more than one gene or gene copy that can complete the pathway. Several genes involved in vitamin biosynthesis are located on symbiont genome plasmids.

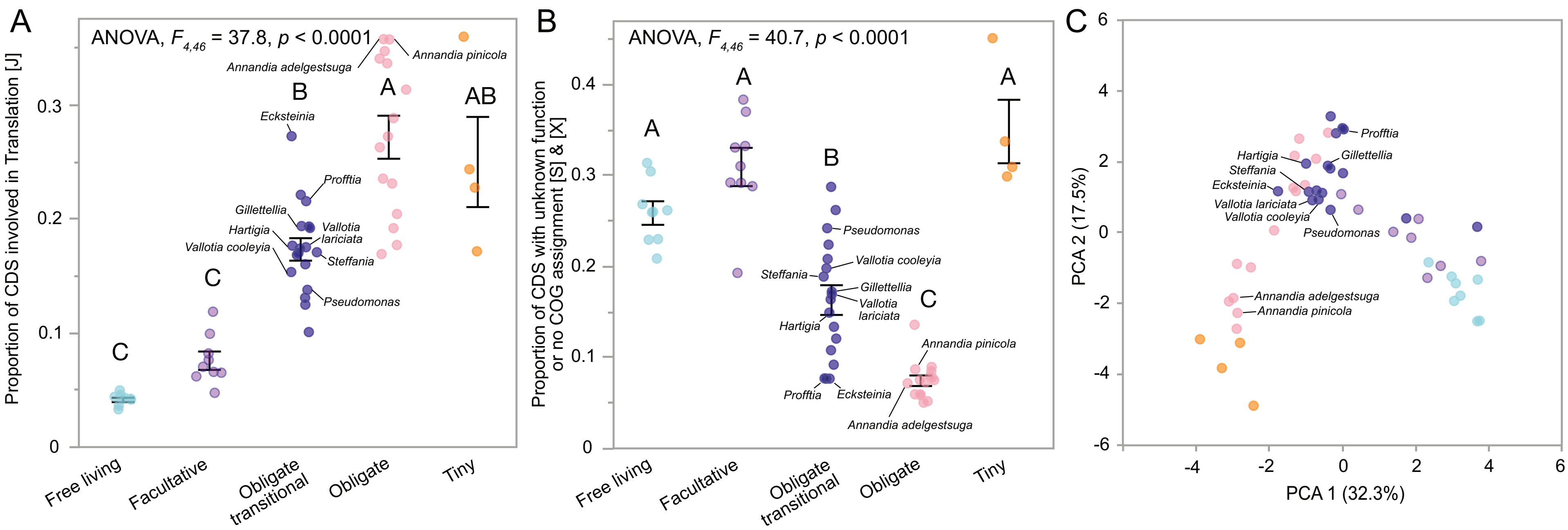

Supplementary Figure 3. Analysis of proportions of CDS belonging to specific COG categories for bacteria that are free-living or insect symbionts in different stages of genome degradation. A. Proportion of CDS involved in COG category J: translation. B. Proportion of CDS involved in COG categories S and X: unknown function or no COG assignment. Error bars indicate standard error of means, and letters above error bars represent differences among means. Principal Components Analysis (C) of variation in proportions of CDS belonging to all COG categories. Bacterial types are colored as for panels (A) and (B). Adelgid symbionts are indicated with text labels.

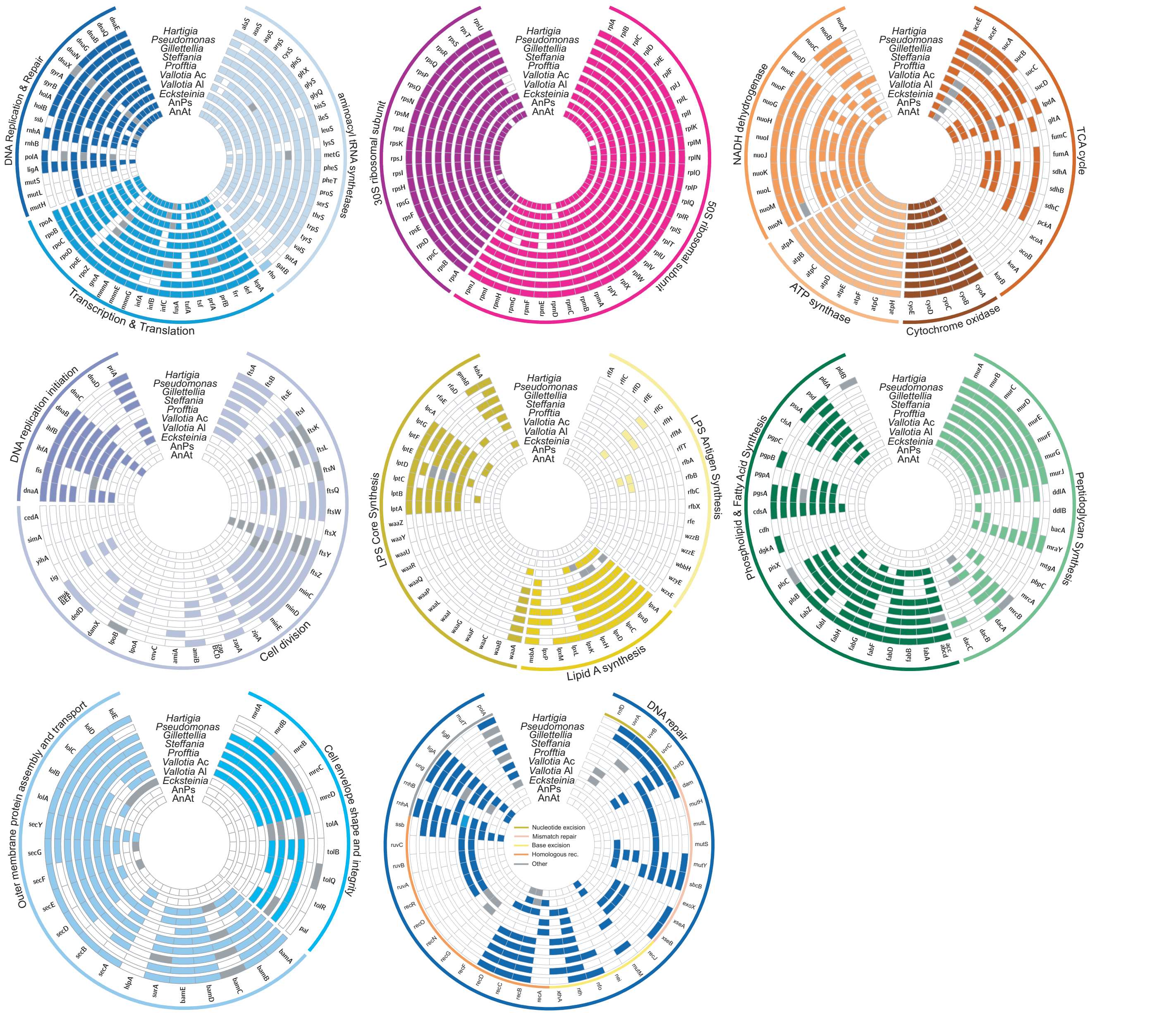

Supplementary Figure 4. Conservation of core genes involved in central cellular processes for adelgid symbiont chromosomes. Colored boxes represent gene presence, unfilled boxes represent gene absence, and gray boxes indicate pseudogenes.

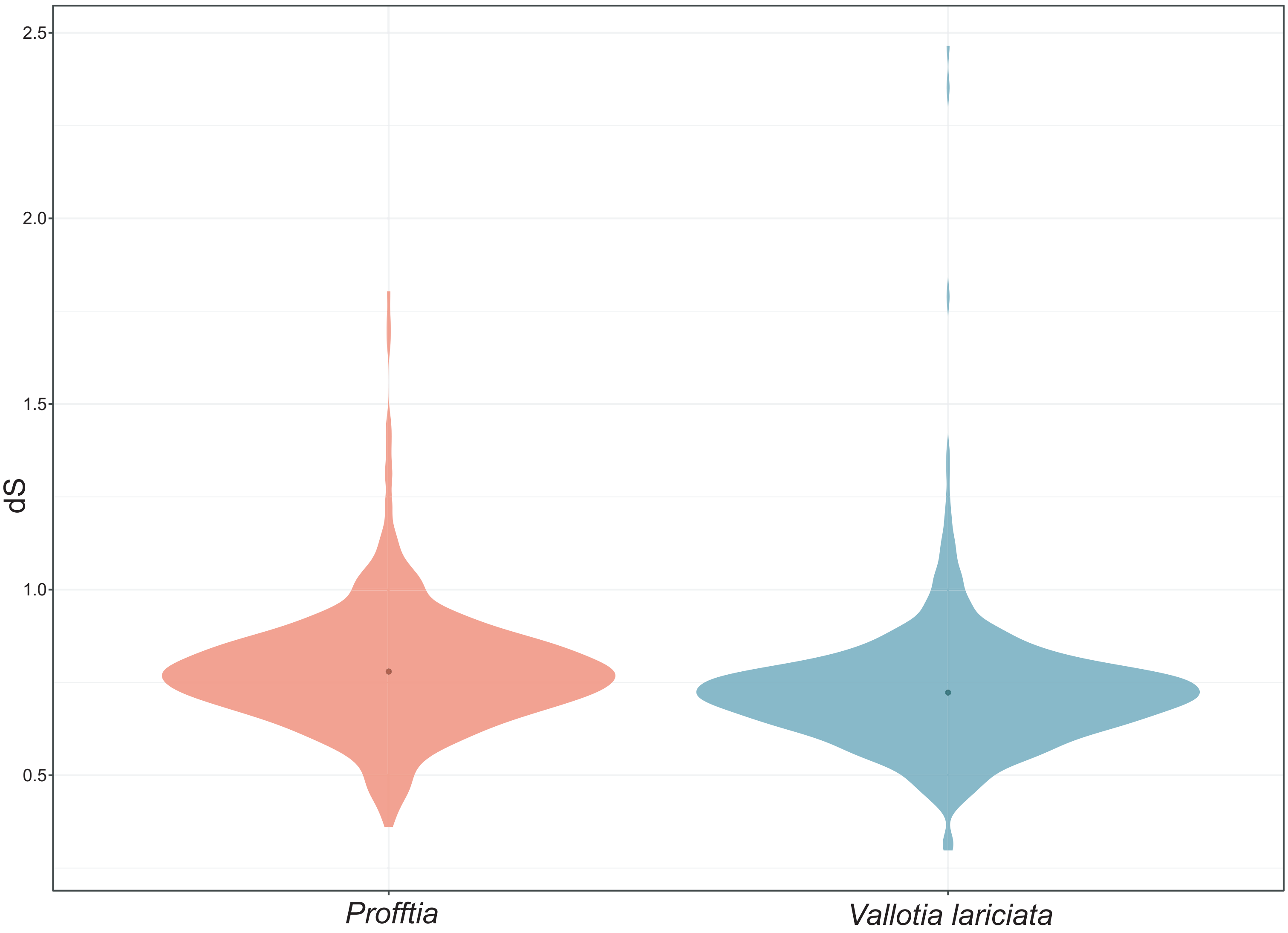

Supplementary Figure 5. Violin plots of the distributions of dS values for pairwise comparisons of orthologous genes in *A. lariciatus* and *A. kitamiensis* symbionts. Mean values are indicated by points and are 0.78 and 0.72 for *Profftia* and *Vallotia*, respectively.
